# Chagas Disease: “The New HIV/AIDS of the Americas”

**DOI:** 10.1371/journal.pntd.0001498

**Published:** 2012-05-29

**Authors:** Peter J. Hotez, Eric Dumonteil, Laila Woc-Colburn, Jose A. Serpa, Sarah Bezek, Morven S. Edwards, Camden J. Hallmark, Laura W. Musselwhite, Benjamin J. Flink, Maria Elena Bottazzi

**Affiliations:** 1 Departments of Pediatrics and Molecular Virology & Microbiology, and Sabin Vaccine Institute and Texas Children's Center for Vaccine Development, Baylor College of Medicine, Houston, Texas, United States of America; 2 National School of Tropical Medicine, Baylor College of Medicine, Houston, Texas, United States of America; 3 Laboratorio de Parasitologia, Centro de Investigaciones Regionales “Dr Hideyo Noguchi”, Universidad Autonoma de Yucatan, Merida, Yucatan, Mexico; 4 Section of Infectious Diseases, Department of Internal Medicine, Baylor College of Medicine, Houston, Texas, United States of America; 5 Department of Emergency Medicine, Baylor College of Medicine, Houston, Texas, United States of America; 6 Section of Infectious Diseases, Department of Pediatrics, Baylor College of Medicine, Houston, Texas, United States of America; 7 Houston Department of Health and Human Services, Houston, Texas, United States of America; 8 Duke University School of Medicine, Durham, North Carolina, United States of America

*Endemic Chagas disease has emerged as an important health disparity in the Americas. As a result, we face a situation in both Latin America and the US that bears a resemblance to the early years of the HIV/AIDS pandemic.*

Neglected tropical diseases (NTDs) are among the most common conditions afflicting the estimated 99 million people who live on less than US$2 per day in the Latin American and Caribbean (LAC) region [1]. Almost all of the “bottom 100 million” living in the Americas suffer from at least one NTD [1], and according to some estimates, the NTDs cause a burden of disease in the LAC region that closely approximates or even exceeds that resulting from HIV/AIDS [2]. Chagas disease (American trypanosomiasis) is a vector-borne disease and a leading cause of the deaths and disability-adjusted life years (DALYs) lost that result from NTDs in the LAC region [2]. With approximately 10 million people living with Chagas disease, this condition is one of the most common NTDs affecting the bottom 100 million in the region, a prevalence exceeded only by hookworm and other soil-transmitted helminth infections [1], [2]. Moreover, among the NTDs in the Americas, Chagas disease ranks near the top in terms of annual deaths and DALYs lost [1], [2].

While most of the world's cases of Chagas disease occur in the LAC region, there is increasing recognition that many people with *Trypanosoma cruzi* infection also live in the US and Europe [3]. In practical terms, the “globalization” of Chagas translates to up to 1 million cases in the US alone, with an especially high burden of disease in Texas and along the Gulf coast [4], [5], although other estimates suggest that there are approximately 300,000 cases in the US [6], in addition to thousands of cases documented in Canada, Europe, Australia, and Japan [3]. Among those living with Chagas disease around the world today, 20%–30% (roughly 2–3 million people) are either currently suffering from Chagasic cardiomyopathy or will develop this clinical sequela [7]. Chagasic cardiomyopathy is a highly debilitating condition characterized by cardiac arrhythmias, heart failure, and risk of sudden death from ventricular fibrillation or tachycardia or thromboembolic events [7]. Another estimate suggests that up to 5.4 million people living today will develop Chagasic cardiomyopathy [8], [9]. Damage to the gastrointestinal tract can also produce debilitating megaesophagus and megacolon [7].

There are a number of striking similarities between people living with Chagas disease and people living with HIV/AIDS, particularly for those with HIV/AIDS who contracted the disease in the first two decades of the HIV/AIDS epidemic. Both diseases are health disparities, disproportionately affecting people living in poverty [1], [2]. Both are chronic conditions requiring prolonged treatment courses: a lifetime of antiretroviral therapy for HIV/AIDS patients, and one to three months of therapy for those with Chagas disease [7]. Treatment for HIV/AIDS is lifesaving, although it seldom if ever results in cure, while for Chagas disease, the treatment has proven efficacy only for the acute stages of the infection or in children up to 12 years of age during the early chronic phase of the infection [10]. For both diseases the treatment is expensive—in the case of Chagas disease, the expected cost of treatment per patient year is $1,028, with lifetime costs averaging $11,619 per patient [11]. Exacerbating costs, Chagas disease itself is a serious opportunistic infection of people living with HIV/AIDS, and is associated with meningoencephalitis, cerebral lesions, and high mortality [7]. As with patients in the first two decades of the HIV/AIDS epidemic, most patients with Chagas disease do not have access to health care facilities. A recent analysis indicates that many patients do not have access to the essential medicines for Chagas disease, in particular, the first line of therapy, the drug benznidazole [12]. According to Médecins Sans Frontières (MSF, Doctors Without Borders), many highly endemic countries, including Paraguay and Bolivia, face acute shortages of benznidazole, forcing thousands of newly diagnosed patients to postpone treatment [12]. Both diseases are also highly stigmatizing, a feature that for Chagas disease further complicates access to benznidazole and other essential medicines, as well as access to serodiagnosis and medical counseling. For some individuals with *T. cruzi* living in the US, immigration status presents an additional challenge to seeking care and prevention services. Just as stigma due to sexual orientation has been a barrier to HIV care and prevention, especially in the beginning of the epidemic, immigration status may function as a deterrent to Chagas disease care and prevention.

Based on the assertions outlined above—the chronic morbidities and high mortalities, the prolonged and expensive treatment courses, the lack of therapeutic options, and barriers to access to essential medicines—a patient living with Chagas disease faces formidable challenges that resemble those faced by someone living with HIV/AIDS, especially the challenges that occurred in the early years of the HIV/AIDS epidemic. Shown in Table 1 is a quantitative comparison between people living with Chagas disease [2], [4], [6], [13], [14] and those living with HIV/AIDS in the LAC region and the Americas [13]–[17]. Briefly, the roughly 10 million people living with Chagas disease (including 2–5 million individuals with Chagasic cardiomyopathy) is comparable in number to the 1.6 million people living with HIV/AIDS in the LAC region and the 1–2 million living with HIV/AIDS in North America (including Mexico). However, based on current estimates, both the number of annual DALYs lost and the attributed deaths are about five times higher for HIV/AIDS. The morbidity and mortality estimates for both Chagas disease and HIV/AIDS are undergoing revision by the University of Washington Institute of Health Metrics and Evaluation.

**Table 1:** Comparison of Chagas disease versus HIV/AIDS.

Disease	Estimated Number of Cases in LAC [Reference]	Estimated Number of Cases in North America [Reference]	Estimated DALYs in the Americas [Reference]	Estimated Annual Deaths in the Americas [Reference]	Countries Most Affected [Reference]
Chagas disease	8–9 million (2–5 million cases of Chagasic cardiomyopathy) [2]	0.3–1.0 million in the US [4], [6]	0.7 million [13]	14,000 [14]	Bolivia, Mexico, Colombia, Central America [2]
HIV/AIDS	1.6 million [15], [16]	2.3 million^a^ [17]	3.2 million [13]	105,000^a^ [15]–[17]	Brazil, Mexico, Colombia, United States [15]–[17]

a The UNAIDS data for North America also includes Western and Central Europe. According to the US Centers for Disease Control and Prevention, at the end of 2008 an estimated 1.2 million people were living with HIV in the US [40].

There are additional parallels. Chagas disease has emerged as an important blood transfusion–related risk throughout the Americas just as HIV/AIDS did in the early 1980s, prior to the implementation of widespread blood screening and testing [18]–[20]. Moreover, mother-to-child transmission leading to congenital Chagas disease and other adverse neonatal outcomes is increasingly recognized [21]–[24] (Table 2). Both congenital Chagas disease and HIV/AIDS have a recognized clinical syndrome [21], [25], with adverse birth outcomes as well as deleterious maternal effects in pregnancy [22], [23], [26]. During pregnancy, the rate of vertical transmission of *T. cruzi* infection is approximately 5% (although some investigators believe the rate could be as high as 10%), whereas it is 15%–40% for untreated HIV/AIDS [27] and 1%–2% for mothers who receive antiretroviral therapy [28]. The Pan American Health Organization estimates that there are over 14,000 cases of congenital Chagas disease in Latin America [29], with 2,000 newborns infected annually in North America alone [24], compared to 36,000 pediatric HIV/AIDS cases in Latin America [15].

**Table 2:** Comparison of congenital Chagas disease and HIV/AIDS.

Category	Congenital Chagas Disease [Reference]	Congenital HIV/AIDS [Reference]
Major clinical features	Hepatosplenomegaly, hydrops, neonatal death [21].	Failure to thrive, opportunistic infections, lymphocytic interstitial pneumonitis, developmental delays [25].
Effects in pregnancy	Pregnancy enhances parasitemia [22]. Increased risk of miscarriage, preterm birth, neonatal infection [23].	Increased spontaneous abortion, chorioamnionitis, low birth weight, prematurity [26].
Vertical transmission	Vertical transmission of 5% from seropositive mothers [23], although some investigators believe the transmission rate may be higher.	Vertical transmission of 15%–40% without maternal treatment [27]. Vertical transmission of 1%–2% on antiretroviral treatment [28].
Number of cases	14,000 cases in Latin America [29]. 40,000 pregnant women in North America, 2,000 newborns are infected annually [24].	36,000 pediatric HIV/AIDS cases in Central and South America [15].

It is only appropriate to point out that there are important differences between Chagas disease and HIV/AIDS. Whereas HIV/AIDS is almost always a fatal condition in the absence of antiviral therapy, up to 70%–80% of people with Chagas disease do not progress to cardiomyopathy. Moreover, Chagas disease is a true NTD and there is a paucity of anti-protozoan drugs available for this condition, whereas HIV/AIDS can no longer be considered neglected in this sense as there is an armamentarium of antiretroviral drugs currently available (although for both conditions, patients in developing countries suffer from lack of access to essential medicines). As another contrast between the two diseases, there is also evidence for oral transmission of Chagas through food contamination in the Amazon basin [30].

Over the last three decades of the global HIV/AIDS pandemic, an aggressive and committed activist community has achieved success in promoting widespread access to antiretroviral drugs in developed and developing countries. As a result, at present millions of people living with HIV/AIDS receive antiretroviral therapy, and pediatric HIV/AIDS has been nearly eliminated as a public health problem in the US [31]. Patient advocacy and global efforts to promote access to benznidazole and other therapies for Chagas disease, on the other hand, are at a much earlier stage. In the last decade, MSF has launched efforts to screen more than 80,000 people in Bolivia, Colombia, Guatemala, Honduras, Nicaragua, and Paraguay, having diagnosed and treated more than 6,000 and 4,000 individuals, respectively [12]. Other non-governmental organizations have also been engaged in Chagas disease treatments. Of great concern is a looming shortage of benznidazole, as well as the over-reliance on a single drug manufacturer, and inadequate international efforts to organize global production and distribution of the drug in Latin America [12]. Nifurtimox, another drug commonly used in the treatment of Chagas disease, should also be made available for the LAC region.

In parallel with global advocacy efforts, expansion of operational research activities is crucial to optimize the efficacy of existing control and elimination efforts, including the testing of more field-adapted tools [12]. There are also requirements to expand vector control activities and health education in the communities affected by Chagas disease, in addition to providing training for local health care providers in endemic areas [12]. The importance of a research and development agenda to develop new and improved Chagas disease drugs cannot be overlooked. As noted above, both antitrypanosomal drugs used for the treatment of Chagas disease, benznidazole and nifurtimox, require prolonged and expensive treatment courses; moreover, the efficacy of either drug for the treatment of late chronic infection and Chagasic cardiomyopathy still remains uncertain and unproven [32], [33]. Toxicities and the frequency of side effects of both medicines frequently require patients to interrupt or halt treatments, and both drugs are contraindicated in pregnancy [34]–[36]. In response to this crisis, the product development partnership (PDP) Drugs for Neglected Diseases initiative (DNDi) is accelerating the development of new Chagas disease drugs in the non-profit sector, in parallel with a small group of academic laboratories that are pursuing several drug targets [37], [38]. Finally, just as the International AIDS Vaccine Initiative (IAVI) PDP is developing several experimental HIV/AIDS vaccines, the Sabin Vaccine Institute PDP is accelerating the development of a new therapeutic Chagas disease vaccine in collaboration with several key universities and public sector biotechnology and manufacturing institutions in Mexico [1]. In further support of this research, a recent analysis by Lee and his colleagues confirms the potential cost effectiveness of Chagas disease vaccines [39].

Stark similarities exist between today's global Chagas disease epidemic and the first two decades of the HIV/AIDS epidemic. This translates into a humanitarian catastrophe for the poorest people in the Americas and elsewhere. This perceptible health disparity demands urgent attention by global health policy makers to prioritize Chagas disease and develop a comprehensive strategy for control and elimination efforts, blood screening and point-of-care testing, maternal and child interventions, health education, and parallel research and development. Successfully addressing the vast burden of Chagas disease will require overcoming the current lack of available drugs, together with expanding vector control strategies and developing new and innovative control tools. To this end, eliminating Chagas disease will require a commitment from international health agencies, governments of the disease-endemic countries, PDPs, and patient advocacy groups.
